# Digital silence: the psychological impact of being shadow banned on mental health and self-perception

**DOI:** 10.3389/fpsyg.2025.1659272

**Published:** 2025-10-07

**Authors:** Sobi Thomas, Paul Manalil

**Affiliations:** ^1^School of Social Science, Arts and Humanities, Lincoln University College, Petaling Jaya, Malaysia; ^2^Department of Communication and Media Studies, Marian College Kuttikkanam, Kuttikkanam, India

**Keywords:** shadow banning, algorithmic invisibility, self-perception, digital silence, mental health, media psychology, online validation, emotional regulation

## 1 Introduction: when the feed goes quiet

Imagine that you publish a well-thought-out post, photo, or video—only to watch it silently sink into obscurity. No likes, no comments, no shares. At first, you can brush it off as a fluke or a bad content day. But for many of us, specifically for those who are on active social media platforms like Instagram, Tiktok, or X (previously Twitter), Shadow banning could be the primarily cause resulting in their silence. Shadow banning could be defined as an algorithmic hiding in which the content is quietly de-amplified without no indication (Liu et al., 2023). To differentiate overt censorship from shadow banning, it is an act with a conscious face whereas shadow banning is invisible and creates a sense of social erasure that could potential result in emotional disorientation and psychological distress. Recently, research studies have begun emphasizing the importance to recognize the shadow banning not only as a technical limitation but also on a broader spectrum on digital exclusion and algorithmic marginalization (Delmonaco et al., 2024).

In this paper, we examine shadow banning more as an intensely subjective psycho-existential phenomenon rather than as a technical bug or policy enforcement strategy. Findings of this study show that Shadow banning emotionally affect the self-concept leading to disruptions in digital social feedbacks. The individuals are therefore compelled to rely for validation identification, reinforcement, and social inclusion. This study did a detailed analysis of the literature in media psychology and theories of emotional and digital behavior, and concludes that non-transparency of the social media platforms causes distress of individuals, and it needs to be addressed urgently.

## 2 Understanding shadow banning and its affective mechanism

Shadow banning also known as Stealth banning, silently prevents or restricts a user's reach in the social media platforms. It is a kind of algorithmic suppression without suspending the account. Unaware of the invisibility of the post in the community the user till continues posting, but the message never appears in search results, hashtags, or regular feeds, leading to decreased engagement. These users are, in fact, speaking to a void. This digital silence can be described as a vocal message within the social media economy.

Feedback is a sustainable rejuvenating factor of the online platforms. The activating responses through “likes, comments, reposts and follows” are emotional assets which indicates self-affirmation. These validations cannot be ignored as they activate neural centres which releases dopamine. When these signals vanish into thin air with no indication of why the users feel lost, rejected and struggle with cognitive dissonance (Politte-Corn et al., 2024). Am I ignored? Is my content awful? Have I done something inappropriate? The withdrawal from engagement is a psychological riddle that upsets the self-worth. From a psychological standpoint, this dynamic activates the mesolimbic dopamine system, reinforcing the role of social affirmation in self-perception (Cross et al., 2025). Cognitive dissonance arises when one's self-image as a socially engaged digital citizen clashes with unexplained algorithmic suppression. A qualitative analysis using Impression Management Theory and Cognitive Dissonance Theory found that teens experience dissonance when their social media presence conflicts with their real-world identity, often leading to discomfort and eventual withdrawal from online activity (Marta and Miletresia, 2022).

**Figure 1:**
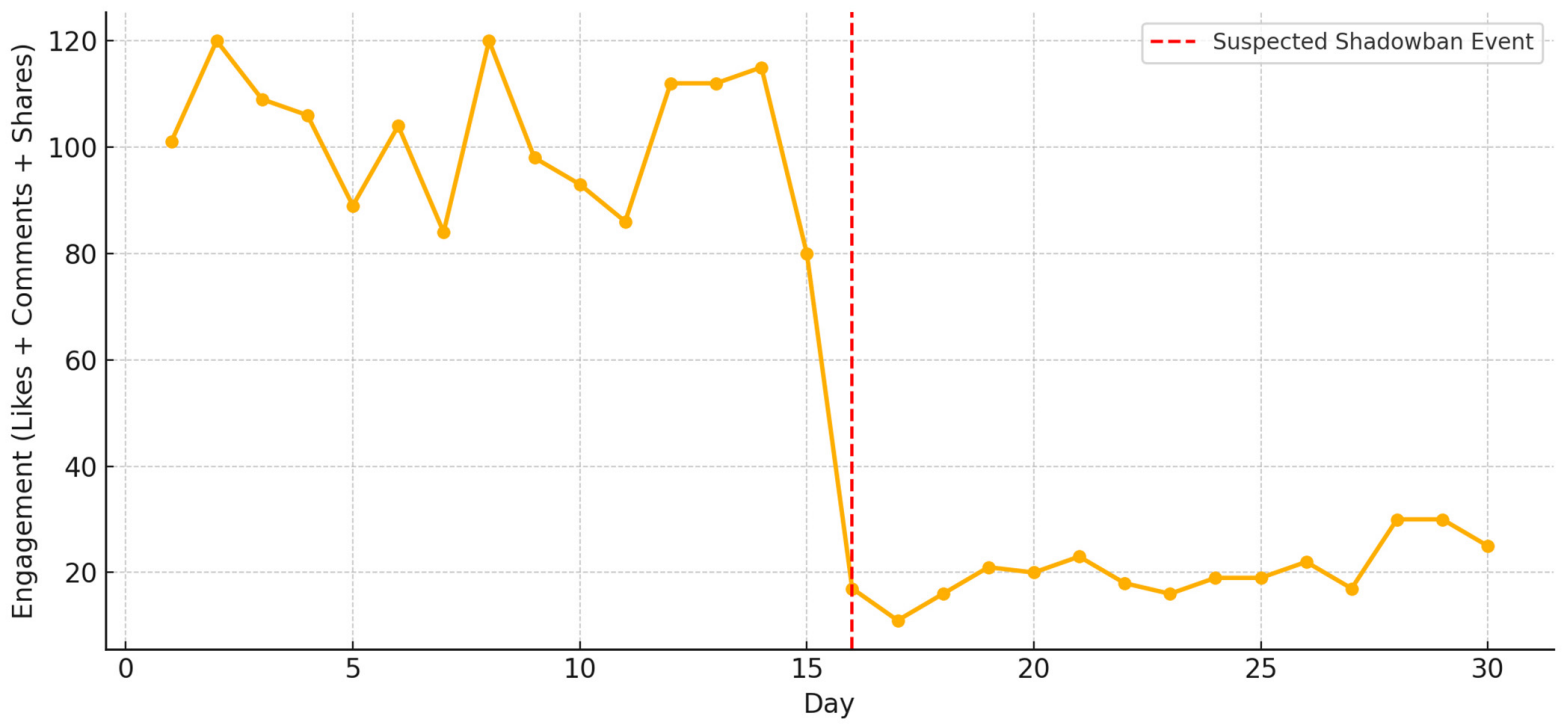
Sudden drop in engagement metrics after suspected shadowban event. This figure is based on a composite of self-reported case patterns drawn from user forums and anecdotal experiences. It is presented illustratively to depict a typical engagement trajectory following suspected shadow banning.

The line graph in the Figure 1 illustrates a noticeable decline in user engagement (likes, comments, shares) following a suspected shadow ban. The data is based on user-reported case studies, showing normal interaction patterns in the days prior (Days 1–15), followed by a significant drop post-event (Days 16–30). This pattern exemplifies the experience of “digital silence,” where content visibility is algorithmically suppressed without user notification, leading to emotional confusion and self-doubt. While this visual is based on informal reports and lacks formal statistical validation, it reflects a recurring pattern documented across multiple user narratives.

## 3 Emotional dysregulation and self-doubt in a platformed identity

Online, the identity is not just described—it is staged and legitimated in the public sphere. The self is algorithmically discernible, constituted with interaction metrics and validation from followers. When a user is shadow banned, they are systematically excluded from the social world. The shock invisibility disrupts emotional regulatory protocols and can induce depression symptoms, anxiety, and compulsive checking of content behaviors (Wikman et al., 2022).

The concerns of social exclusion were studied by media psychologists in recent years and their findings focus on the “indefiniteness” of shadow banning. The users were not told about the banning and the indefinite nature of such banning. The individuals quite often doubt their perception of reality and the emotional cost of exclusion from the social media platforms in high, particularly for creators of activist postings, often associated with political assertions of minority users (Powers et al., 2013). The freedom of expression of such communities is infringed through shadow banning. As no one is held responsible it makes emotional recuperation more difficult. The lack of feedback from the social media platforms, particularly among the users result in emotional dysregulation or a difficulty in managing emotional responses in accordance with the contextual demands (Rogier et al., 2024).

For the individual users the silent platforms are a failure of their own. Such instances ultimately lead to detrimental thinking patterns like repeated checking of the reach of the posts, resubmission and republishing of posts or immerse in self-critical thinking. It not only frustrates but psychologically damage the user (Da Silva Pinho et al., 2024).

## 4 Algorithmic inequality and emotional toll of shadow banning

The impact of shadow banning is not equally affected. The posts which are themed on sexuality, racial disturbances, social activism or body-positive are invariably censored. When these posts are not against the rules it reaches the users (Foster et al., 2021). There are many inherent structural inequalities due to algorithmic governance.

The subaltern and fringe groups in the society who are considered marginalised population always feel that their visibility is conditional and carefully crafted. The content provides belonging to queer and fat rights organisers negotiate their own space in the media for interactions and survival protests (Escobar-Viera et al., 2023). Some minority groups like queer had modest following on Instagram, but later when they discussed other general social issues there was sharp drop in views on all subsequent posts. The digital silencing occurs without formal notices and eventually it leads to distress and a temporary social media hiatus; an emotional erasure that sustains systemic silencing. It is a shame on individuals who feel that invisibility is a personal failure than a structure defect of media. As Covin (2021) emphasizes, shadow banning can lead to “unseen shame,” where users privately struggle with feelings of inadequacy, internalizing their online invisibility as a personal failing, despite the lack of explicit criticism from others.

Recent studies on digital exclusion reveal that algorithmic decisions can perpetuate existing social inequalities online, leaving users feeling unfairly penalized for their identity or views. The constant pressure to create content, coupled with the algorithm's silent devaluation of their voice, can be exhausting (Nair et al., 2024).

## 5 Shadow banning stems from inherent ambiguity?

When uncertainty increases anxiety and causes psychological distress it eventually leads to repetitive negative thoughts and thereby aggravate mental health concerns (Altan-Atalay et al., 2023).

The shadow banned users repeatedly fall into uncertainties even as they continue the futile exercise of selecting hashtags. The emotional exhaustion produces helplessness and bewilderment. The ambiguity linked to the posting in the social media can impact on trans-diagnostic factors linked to anxiety disorders and obsessive rumination. It renders the users more susceptible to distress (Pinciotti et al., 2021). The intolerant situation caused by uncertainty compels the users to quit the site because silence became unsustainable psychologically. Covin (2021) notes that this hidden shame in digital environments rarely has a reintegrated function. It isolates the user and increases his or her isolation. This corresponds with Jochan and Banerjee (2021) argument that shame in digital environments rarely has a reintegrated function; instead, it isolates the individual and deepens alienation.

The obscure element in the shadow banning process disrupts digital trust. Though the social media platforms claim freedom of expression they involve in stealth moderation that facilitates self-censorship and self-policing (Wang and Kim, 2023). This phenomenon can subtly persuade unwilling users into altering their tone and the themes, which eventually lead to emotional conformity due to prolonged limitation on the freedom of expression. The present study focuses on the urgent need for specific interventions to address the issues of ambiguity and emotional impact of algorithmic governance related to shadow banning. The negative psychological effects are far-reaching and it includes exclusion, shame and loss of trust. The transparency in the process of algorithmic governance and alleviation of deeply emotional and identity related constraints the users face online must be prioritized in finding solutions (Risius and Blasiak, 2024).

## 6 A humane platform design and emotional transparency needed

There is an invisible layer of shame in the social media platforms which highlights not only the fundamental issues of algorithmic transparency, but also the hidden psychological costs, ensuring that design responses attend to both external visibility and internal will-being (Covin, 2021). The social media platforms must acknowledge the damage caused by opaque algorithms and adopt transparent practices to reduce the emotional harm done to the users. If the reasons behind the content moderation decisions are explained the platforms can reduce user anxiety and build trust, creating a more open and reliable online environment (Jansen and Krämer, 2023).

Platforms should design with users' mental health in mind, incorporating features such as notifications, appeal options, and transparent explanations for content visibility. Fair governance demands transparency, due process, and accountability, rather than unexplained penalties (Russ et al., 2014). Openness is not a technical remedy; it is a psycho logical necessity.

The mental health practitioners should include algorithmic exclusion within their conceptual framework of digital trauma (Barton et al., 2023). The sudden invisibility resulting from shadow banning can precipitate profound identity crises and emotional distress. Mental health professionals should be trained to address these concerns. Moreover, media literacy initiatives should extend beyond filter bubbles and misinformation to encompass the emotional consequences of algorithmic silence. Further research is warranted to explore the intersections between online trauma and other digital harms, such as cyberbullying, harassment, and community disintegration, to comprehensively understand the phenomenon's scope and implications (Delmonaco et al., 2024).

Despite being dismissed as conspiracy theories, shadow banning can cause real harm. We need more research that combines platform data, user experiences, and signs of psychological distress to understand the true mental health impact of being algorithmically suppressed online.

## 7 Conclusion: making the invisible visible

Visibility is validation in the social media platforms. Shadow banning turns invisibility into a weapon, and the silent treatment of the feed a tool of emotional coercion. Faith in the platforms erodes, shattering the users' perceptions of the self, and digital neurosis and self-doubt intensify (Van Noordt et al., 2015).

This opinion piece contends that shadow banning transcends content moderation, posing a significant psychological concern. By disrupting emotional regulation, exacerbating social inequalities, and fostering cognitive dissonance, it takes a profound toll on users. To mitigate this, media platforms must prioritize the emotional impact of algorithmic governance, lest users continue to experience silent suffering, overshadowed by both code and emotional distress.

To make the invisible visible is the first step toward justice—technical, social, and psychological. Let that apply not only to content, but to the human costs hidden behind the feed.
